# Potential solutions for manufacture of CAR T cells in cancer immunotherapy

**DOI:** 10.1038/s41467-022-32866-0

**Published:** 2022-09-05

**Authors:** Ulrich Blache, Georg Popp, Anna Dünkel, Ulrike Koehl, Stephan Fricke

**Affiliations:** 1 Fraunhofer Institute for Cell Therapy and Immunology (IZI), Leipzig, Germany; 2 Fraunhofer Cluster of Excellence for Immune-Mediated Disease, Frankfurt, Germany; 3 Institute of Clinical Immunology, University of Leipzig, Leipzig, Germany

**Keywords:** Tumour immunology, Tumour immunology

## Abstract

CAR T cell therapy is an effective cancer treatment, but biological and manufacturing hurdles hamper its broad breakthrough. Although the first step towards automated manufacture of CAR cells has been taken, new technologies are needed to enable the treatment of large patient groups.

## The increasing need to produce autologous CAR T cells for cancer therapy

**Fig. 1:**
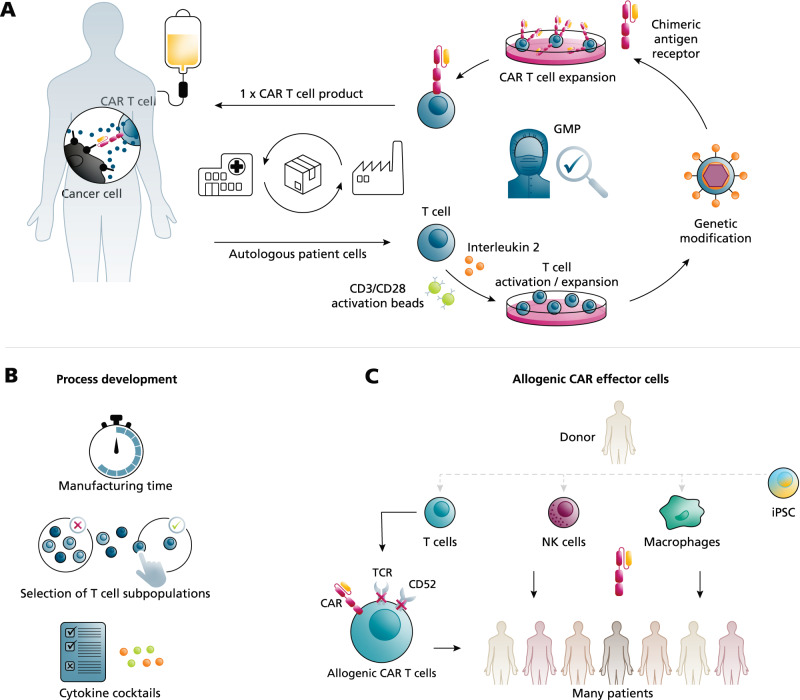
Manufacturing of CAR T cells and biological approaches to increase the availability of CAR immunotherapies. **A** Currently approved autologous chimeric antigen receptor (CAR) T cell immunotherapy is based on a person’s own T cells that are given back to the same patient after ex vivo modification. The production process is performed under Good Manufacturing Practice (GMP) conditions and monitored by several quality controls. **B** Biological approaches to increase CAR T cell quality by process development include the reduction of the manufacturing time, the selection of efficient T cell or CAR T cell subpopulations and the required cytokine combinations in order to avoid exhausted cells. **C** For allogenic CAR immunotherapy, different immune effector cells are obtained from healthy donors and can be given to a large cohort of patients. To enable allogenic CAR T cells, depletion of their T cell receptor (TCR) and the CD52 molecule is performed before they are further modified with the CAR. Other promising immune effector cells are natural killer (NK) cells or macrophages. To obtain cells for allogenic CAR cells, mature healthy donor cells could be derived from induced pluripotent stem cells (iPSC) in the future.

Harnessing the capacity of the patient’s own immune system to fight malignant cells is the basis of adoptive cancer immunotherapy. Treatment with T cells genetically equipped with chimeric antigen receptors (CAR) against CD19 has led to impressive remission rates in cancer patients and, up to date, five CAR T cell products are market approved for third-line therapy of hematological malignancies. Furthermore, based on new phase III results and first FDA-approvals, CAR T cell treatment will also become a second-line care option^1^. Currently, the main research focus is on overcoming the molecular limitations of CAR T cell immunotherapy, including adverse effects, immunosuppression in the tumor microenvironment and the resistance of solid cancers^2,3^. The potential of adoptive cancer immunotherapy is currently being investigated in hundreds of clinical trials – many of them using CAR T cells^4^. It is therefore expected that the range of approved clinical therapies will grow continuously and that the number of eligible patients will strongly increase. This is of paramount importance if, in the future, also solid cancers are to be treated using CAR T cells because solid cancers are more frequent compared with hematological malignancies. However, the demand for CAR T cells and related adoptive immunotherapies will likely exceed the current production capacity. To unleash the full potential of adoptive immunotherapy, there is a pressing need to leverage new biological and manufacturing technologies that together will ensure the availability of CAR T cell products. At present, only autologous CAR T cells are market approved and their manufacture is complex with only a few centers worldwide able to execute centralized production. After the patient’s leukapheresis material has arrived at the production facility, T cells are stimulated, transduced by viral vectors encoding for the CAR receptor, expanded and cryopreserved under Good Manufacturing Practices (GMP) conditions, and then shipped back to the original patient (see Fig. 1A). The manufacturing processes are largely performed manually or in a semi-automated manner, which not only causes low throughput but also contributes to product variability and very high costs. Therefore, several process parameters are being investigated to optimize CAR T cell production (Fig. 1B). Strong efforts by both the pharmaceutical industry and academic institutions have significantly shortened the manufacturing time from approximately 14 days down to a few days or, even, 1 day^5^. Because the patient’s individual leukapheresis cells and subsequent CAR T cell subpopulations influence clinical success^6–10^, manufacturing protocols and cytokine cocktails could be refined to enrich or select favorable and non-exhausted T cell subpopulations. In the future, further biological research, process development and hardware technologies will optimize the manufacturing of autologous CAR T cells, but throughput limitations will remain with regard to the personalized, that is individualized, setting.

## Allogenic CAR effector cells for off-the-shelf products

A very promising solution to increase the availability of CAR cell products is allogenic immunotherapy. Here, instead of using autologous patient material, cells from healthy donors serve as source material for CAR cell production (Fig. 1C). The benefits of allogenic CAR immunotherapy are that clinical application can be strongly accelerated by having CAR cells available off-the-shelf and that many more patients can be treated. In addition, producing large batches of allogenic CAR cell products has the potential to lower the required human resources and thereby the manufacturing costs per product, but very careful calculations are needed to draw final conclusions. The number of patients treated from one allogenic batch depends on protocols and technologies for the production of efficient and persistent CAR cells at high yield. For CAR T cells, allogenic approaches are facing fundamental problems that are caused by the immunological barrier. One of the strategies used to avoid alloimmune rejection, and Graft versus Host Disease (GvHD), is to disrupt the T cell receptor (TCR) by genome-editing, leading to universal CAR T cells. Early clinical studies with TCR-negative anti-CD19 CAR T cells (derived from three different donors) in 21 patients show that allogenic CAR T cells can induce remissions in leukemia patients, of which only very few developed mild GvHD^11^. Alternatively, non-T immune effector cells such as natural killer (NK) cells can be used for allogenic CAR immunotherapy without the need for multiple genetic manipulations because NK cells hardly cause GvHD. For example, in a phase I/II trial, anti-CD19 CAR-NK cells derived from one cord blood have been reported to be efficient in 11 patients suffering from lymphoma or leukemia^12^. Furthermore, also macrophages, phagocytotic cells from the innate immune system, are being explored for CAR immunotherapy^13^. Although allogenic immune effector cells are derived from healthy donors, generating high cell numbers with adult cells is a limitation for allogenic CAR immunotherapies at large-scale, which could be solved in the future by using induced pluripotent stem (iPS) cells. Yet, the time allogenic T and NK cells persist in vivo will likely always be much shorter than the decade-term persistence observed for autologous CAR T cells^14^ and multiple infusions of allogenic CAR effector cells might be needed, making efficient manufacturing solutions more important.

## Automated manufacturing to increase the availability of CAR immunotherapies

**Fig. 2:**
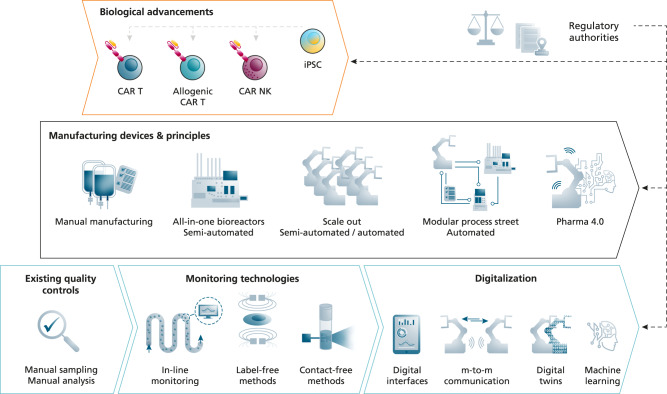
Technological innovations for automated cell manufacturing. Novel manufacturing devices and principles to enable automated CAR cell production. CAR T cells are often produced manually but first semi-automated all-in-one bioreactors are available for the GMP compliant cell manufacturing. For a high-throughput production of autologous products, one solution is using parallelized manufacturing devices. Another principle is connecting different manufacturing devices performing specific sub-processes of the CAR cell manufacturing automatically in process streets. For full automation, these advancements in manufacturing require novel engineering solutions in the fields of real time monitoring and in-line quality control technologies (non-invasive, label-free, contact-free). In addition, automated high-throughput manufacturing of CAR cell products needs the implementation of real-time capable data processing systems, the digital communication of all involved devices and digital representatives of the system (digital twins). All these technical innovations must be closely co-developed and implemented with respect to the biological advancements in CAR immunotherapy and with the requirements of regulatory agencies.

The future demands for CAR-engineered cell products will likely exceed the product number that can be reached by refining current manufacturing protocols and exploiting allogenic CAR effector cells alone. Only a fraction of patients could benefit from adoptive immunotherapy if high-quality clinical-grade CAR cells cannot be manufactured in large numbers. Therefore, alongside with biological innovations, novel production technologies must be developed and implemented to enable a significant increase in product number (see Fig. 2). In the automotive industry, the food industry and also in the classical pharmaceutical industry, automated production solutions are omnipresent, but for manufacturing clinical-grade cells they are still in their infancy. For the GMP-compatible production of CAR T cells, the first semi-automated cell processing systems are on the market and are used for the production of autologous CAR T cells. These bioreactor devices allow cell selection, stimulation, transduction and expansion in a closed system and their feasibility to produce CAR T and NK cells has been shown in a series of studies^15–18^. The interest for producing CAR T cells in a closed system is growing and several new bioreactor systems are being developed by the biotech industry. However, these all-in-one bioreactor devices produce only one product per run, that is at any given time they are occupied with the CAR T cell manufacture for one single patient. One workaround could be the flexible connection of different devices performing specific sub-processes of the CAR T manufacturing process in an automated process street so that each device can operate at high capacity and material from several patients could be processed in parallel. In the future, such manufacturing streets could benefit from the variety of newly developed biomanufacturing devices, provided connectivity between them is ensured. However, in particular for allogenic off-the-shelf CAR immunotherapies, automated and scalable technologies are to be developed so that hundreds of patients can be treated from one cell batch.

## Monitoring and digitalization technologies for manufacturing automation

CAR T cell manufacture requires extensive quality controls (QCs) to monitor the production process and to approve the final product for application in patients, but currently available all-in-one bioreactor devices have only basic QC integrated (i.e., gas, temperature, and pH value). Currently molecular and cellular QC parameters (e.g., cell viability, cell number, cell identity, purity, or CAR receptor expression) are assessed in samples that are manually obtained and processed, which is very time-consuming excluding also real-time adaptation of the production process. Thus, new manufacturing concepts that rely on full end-to-end automation require smart monitoring technologies. Several solutions for automated measurement of cell number and viability exist, but their integration into automated manufacturing workflows needs to be achieved. Yet for CAR T cell products, analytical methods including flow cytometry and qRT-PCR are required to measure cell identity, gene and protein expression, respectively. But for full automation, process monitoring must occur on-line or in-line with the manufacturing process. Therefore, strong research efforts are needed to integrate existing molecular and cellular QC methods into automated manufacturing routes, for instance by developing microfluidic qRT-PCR or flow cytometry devices that are connected to bioreactor platforms. Furthermore, label-free biophysical methods could optimize the QC process because samples can be analyzed in real-time under minimally invasive conditions. For instance, real-time cell deformability (mechanical) cytometry was recently used for immune cell phenotyping and analysis^19^. Optimally, optical methods that are not only label-free but also contact-free, which makes sample collection completely obsolete, could be used. For instance, Walsh et al. remotely determined the activation status of T cells by using autofluorescence imaging^20^.

In addition to new hardware devices, automated production requires machine-to-machine communication and efficient data processing systems to allow for the adaptation and optimization of production processes in real-time (or close to real-time). The integration of automated production platforms, novel monitoring technologies and advanced data analysis will create a challenging system. To help control this manufacturing system, virtual representation, also known as digital twins, can perform simulations and performance tests based on real-time data. However, to bring digital twin systems beyond conceptual approaches strong efforts are needed, in particular, to create open interfaces between the multiple different devices needed for CAR T cell manufacturing. Importantly, it will be vital to evaluate the regulatory compliance of any advanced manufacturing (or biological) technology early on in the process.

## Conclusions

The rise of adoptive immunotherapy leaves us with an unanswered, but under-researched, question: how to produce sufficient numbers of CAR cell products to treat the growing group of patients? Therefore, automated production solutions are needed for the manufacturing of large numbers of CAR T cell products. However, further manufacturing-related questions remain to be answered. For instance, in all market-approved CAR T cells and in the vast majority of clinical studies the CAR gene is delivered into cells by viral vectors, but viral vector production is complex and expensive itself. Alternative non-viral methods include the use of mRNA technologies or transposons and are already included in first proof of concept clinical trials, but further research is needed to improve their longevity and safety, respectively. Another point of discussion is whether in the future decentralized CAR cell manufacture can complement or even replace the current centralized production. For the future of adoptive immunotherapy, we believe that finding solutions to the existing challenges of producing enough CAR cell products at clinical-grade quality will remain an urgent topic for research and translation because they will ensure the ultimate goal of treating many more cancer patients.
